# Differential Effects of the Factor Structure of the Wechsler Memory Scale-Revised on the Cortical Thickness and Complexity of Patients Aged Over 75 Years in a Memory Clinic Setting

**DOI:** 10.3389/fnagi.2017.00405

**Published:** 2017-12-07

**Authors:** Ryuta Kinno, Azusa Shiromaru, Yukiko Mori, Akinori Futamura, Takeshi Kuroda, Satoshi Yano, Hidetomo Murakami, Kenjiro Ono

**Affiliations:** Division of Neurology, Department of Medicine, Showa University School of Medicine, Tokyo, Japan

**Keywords:** attention, cortical thickness, factor structure, fractal dimension, memory clinic, paired associate memory, recognition memory, WMS-R

## Abstract

The Wechsler Memory Scale-Revised (WMS-R) is one of the internationally well-known batteries for memory assessment in a general memory clinic setting. Several factor structures of the WMS-R for patients aged under 74 have been proposed. However, little is known about the factor structure of the WMS-R for patients aged over 75 years and its neurological significance. Thus, we conducted exploratory factor analysis to determine the factor structure of the WMS-R for patients aged over 75 years in a memory clinic setting. Regional cerebral blood flow (rCBF) was calculated from single-photon emission computed tomography data. Cortical thickness and cortical fractal dimension, as the marker of cortical complexity, were calculated from high resolution magnetic resonance imaging data. We found that the four factors appeared to be the most appropriate solution to the model, including recognition memory, paired associate memory, visual-and-working memory, and attention as factors. Patients with mild cognitive impairments showed significantly higher factor scores for paired associate memory, visual-and-working memory, and attention than patients with Alzheimer's disease. Regarding the neuroimaging data, the factor scores for paired associate memory positively correlated with rCBF in the left pericallosal and hippocampal regions. Moreover, the factor score for paired associate memory showed most robust correlations with the cortical thickness in the limbic system, whereas the factor score for attention correlated with the cortical thickness in the bilateral precuneus. Furthermore, each factor score correlated with the cortical fractal dimension in the bilateral frontotemporal regions. Interestingly, the factor scores for the visual-and-working memory and attention selectively correlated with the cortical fractal dimension in the right posterior cingulate cortex and right precuneus cortex, respectively. These findings demonstrate that recognition memory, paired associate memory, visual-and-working memory, and attention can be crucial factors for interpreting the WMS-R results of elderly patients aged over 75 years in a memory clinic setting. Considering these findings, the results of WMS-R in elderly patients aged over 75 years in a memory clinic setting should be cautiously interpreted.

## Introduction

The Wechsler Memory Scale-Revised (WMS-R), one of the internationally well-known batteries for memory assessment in a general memory clinic setting, addresses many of its predecessors' shortcomings (Wechsler, 1987). Two subsequent revisions, such as WMS-III and WMS-IV, have now been published. Nonetheless, the WMS-R is still often used in many countries because the WMS-R has been translated into several languages, such as German, Japanese, and Portuguese. Indeed, the latest version of Japanese translation is WMS-R. Due to its relatively strong psychometric grounding and representative normative sampling, the WMS-R, which includes 12 subtests (with an additional subtest for information and orientation), will likely obtain a prominent position among the numerous neuropsychology batteries (Loring, 1989). Several factor structures for memory-related subtests have been proposed, such as the two-factor model with general memory and attention, the three-factor model with verbal memory, non-verbal memory, and attention, and the three-factor model with attention, immediate memory, and delayed memory (Bornstein and Chelune, 1988; Roid et al., 1988; Burton et al., 1993). Although the WMS-R has been generally used to assess patients aged between 16 and 74 years, several studies have reported on the validity of using this battery for patients aged over 75 years (Doppelt and Wallace, 1955; Ryan et al., 1990; Ivnik et al., 1992), especially in a memory clinic setting (Iseki et al., 2010; Clark et al., 2013; Hori et al., 2013). However, little is known about the factor structure of the WMS-R for patients aged over 75 years and its neurological significance.

Neuroimaging is one possible method for providing relevant insights. Currently, there are two types of neuroimaging methods available: functional and structural neuroimaging. Brain single-photon emission computed tomography (SPECT) can be used as functional imaging for providing three-dimensional (3D) information on the perfusion and metabolic status of brain tissues (Catafau, 2001). In fact, SPECT is used in a memory clinic setting for supporting diagnoses (Pijnenburg et al., 2008; Morinaga et al., 2010). In addition to functional neuroimaging, structural magnetic resonance imaging (MRI) supports clinical diagnoses in a memory clinic setting by identifying certain patterns of atrophy and vascular damage (Wattjes, 2011). Several techniques for assessing cerebral morphology, such as voxel-based morphometry (Ashburner and Friston, 2000) or cortical pattern matching (Thompson et al., 1998) also exist. Recently, surface-based morphometry (SBM) has been used to examine the relation between cortical thickness and cognitive profiles (Fischl and Dale, 2000; Fjell et al., 2006). As SBM provides more precise measurements (such as the actual thickness of the cortex in mm), it is possible to analyze the correlation between cognitive abilities and depth of the cortex across the entire surface of the brain. Several studies have already demonstrated that cortical thickness assessed by SBM is significantly correlated with the memory function (Ystad et al., 2009; Engvig et al., 2010; Palacios et al., 2013). Thus, these functional and anatomical neuroimaging techniques can be used to assess the neurological significance of factor structures of the WMS-R.

SBM can be used to estimate not only cortical thickness but also cortical fractal dimension as the marker of cortical complexity (King et al., 2009). The fractal dimension (the estimate of the topological complexity of an object) has been proposed as a potential surrogate marker of the degree of brain damage in several psychiatric and neurological alterations, due to its sensitivity in detecting brain changes, including those in pathological cerebral aging (Di Ieva et al., 2015), attention deficit hyperactivity disorder (Li et al., 2007), and dyslexic adolescents (Sandu et al., 2008). It is also known that changes in the folding area, in addition to sulcal depth and cortical thickness, have significant effects on the cortical fractal dimension among normal adults (Im et al., 2006). Considering these neurological effects, cortical complexity may be associated with the neurological significance of factor structures of the WMS-R.

This study aimed to clarify the factor structure of the WMS-R and its neurological significance for patients aged over 75 years in a memory clinic setting. For this purpose, exploratory factor analysis (EFA) was performed to determine the factor structure of the WMS-R. Further, we examined regional cerebral blood flow (rCBF), cortical thickness, and cortical fractal dimension, as neuroimaging features. Previous studies have demonstrated age-related changes in memory-related subtests, such as visual reproduction, visual paired associates memory, verbal paired associates memory, and logical memory (Cullum et al., 1990; Kawano et al., 2013). Therefore, this study posits that the factor structure of the WMS-R in patients aged over 75 years can differ from that in patients aged under this age bracket. The findings imply that the WMS-R results can be used to assess patients aged over 75 years in a memory clinic setting.

## Materials and methods

### Participants

The participants of our study were 50 elderly patients [age: 82.8 ± 4.88 (75–93); 21 males and 29 females; education (year): 12.3 ± 2.71] who had consulted at the memory clinic of the Division of Neurology in the Department of Medicine at the Showa University School of Medicine because of subjective memory complaints. The following four conditions comprised the inclusion criteria for the present study: (1) right-handedness; (2) no history of neurological and neuropsychiatric diseases, including cerebrovascular disease; (3) completion of the WMS-R; and (4) no medical problems related to MRI acquisition. For evaluating the usefulness of the WMS-R in a memory clinic setting, all the patients who met the criteria were examined, regardless of their disease profiles. More specifically, there were 23 patients with Alzheimer's disease (AD), 14 with mild cognitive impairments (MCI), two with vascular dementia, two with diffuse Lewy body disease (DLB), three with frontotemporal dementia (FTD), and one with idiopathic normal pressure hydrocephalus (iNPH), in addition to five healthy controls. Each diagnosis was based on the following diagnostic criteria: the Diagnostic and Statistical Manual of Mental Disorders, 5th Edition (American Psychiatric Association, 2013); the National Institute on Aging-Alzheimer's Association workgroup for MCI and AD (Albert et al., 2011; Mckhann et al., 2011); the Third Report of the DLB Consortium for DLB (Mckeith et al., 2005); the criteria for vascular dementia from the International Society for Vascular Behavioral and Cognitive Disorders (Sachdev et al., 2014); the International Behavioral Variant Frontotemporal Dementia Criteria for FTD (Rascovsky et al., 2011; Lamarre et al., 2013); and frequently used criteria for iNPH (Relkin et al., 2005). Approval was obtained from the Institutional Review Board of the Showa University School of Medicine. All subjects gave written informed consent in accordance with the Declaration of Helsinki.

### Behavioral data analysis

Behavioral data were analyzed using JMP-pro version 13.0.0 and R version 3.4.2. Our sample size is *n* = 50, which is supposed to be a reasonable absolute minimum for EFA (Sapnas and Zeller, 2002; de Winter et al., 2009). The EFA was based on the correlation matrix of the 12 items of WMS-R subtest (mental control, design memory, logical memory I/II, visual paired associates I/II, verbal paired associates I/II, visual reproduction I/II, digit span, and visual span). Bartlett's Test of Sphericity [$\chi_{\left(66\right)}^{2}$ = 429.33, *p* < 0.001] and the Kaiser–Meyer–Olkin measure of sampling adequacy (MSA = 0.801) provided evidence that the correlation matrix was adequate for factor analysis (Tabachnick and Fidell, 2007). EFA was conducted using maximum likelihood (ML) estimation and promax rotation. Factor loadings >0.35 were considered significant, while the factor scores for each factor were calculated and applied in the following neuroimaging analyses.

### SPECT acquisition and analysis

All the participants were positioned supine with their eyes closed. After an intravenous bolus injection of 600 MBq technetium-^99m^Tc ethyl cysteinate dimer, the projection data was acquired by using a two-headed gamma camera system (ECAM, Siemens, Hoffman Estates, IL). The global CBF was noninvasively measured using the Patlak plot method (Matsuda et al., 1995). rCBF was calculated by using an automated brain perfusion SPECT analyzing program, 3DSRT (Takeuchi et al., 2003). This program performs the anatomic standardization of images by employing statistical parametric mapping (Friston et al., 1995), rCBF quantification using a three-dimensional stereotactic region of interest (ROI) template, and a calculation of CBF. The 636 ROIs were then categorized into 12 segments (i.e., callosomarginal, precentral, central, parietal, angular, temporal, posterior cerebral, pericallosal, lenticular nucleus, thalamus, hippocampus, and cerebellum). In addition, the correlations between the absolute value of rCBF and the factor scores were examined, which were thresholded at corrected *p* < 0.05 with false discovery rate (FDR) correction for each segments.

### MRI data acquisition and preprocessing

The structural MRI scans were conducted on a 1.5 T MR scanner (Magnetom Essenza, Siemens, Germany). The high-resolutionT1-weighted 3D images of the whole brain (144 sagittal slices; 1.0 × 1.0 × 1.25 mm^3^; repetition time = 1,600 ms; echo time = 4.7 ms; flip angle = 15°; field of view = 256 × 256) was acquired for each patient. The structural MRI data preprocessing was performed in a standard manner by using the CAT12 Toolbox (http://dbm.neuro.uni-jena.de/cat/) in SPM12 (Wellcome Trust Centre for Neuroimaging, http://www.fil.ion.ucl.ac.uk/spm/) (Friston et al., 1995), and was implemented on MATLAB software (MathWorks, Natick, Massachusetts, USA). Preprocessing was performed by the CAT 12 Toolbox under the default setting, except when using the East Asian brain template for affine registration. Firstly, all the T1-weighted anatomical images were manually reoriented in order to place the anterior commissure at the origin of the 3D Montreal Neurological Institute (MNI) space. The images were then segmented into gray matter, white matter, and cerebrospinal fluid (Ashburner and Friston, 2005). Next, they were normalized to the MNI space by using a diffeomorphic non-linear registration algorithm (diffeomorphic anatomical registration through exponentiated lie algebra toolbox-DARTEL) (Ashburner, 2007). The final resulting voxel size was 1.5 × 1.5 × 1.5 mm. For quality assurance, the resulting images were checked for homogeneity. As all of the images had high correlation values (>0.85), none of the images were discarded.

### SBM analysis

SBM analysis was performed using the CAT12 Toolbox. In this analysis, a fully automated method was employed to allow for the measurement of cortical thickness and reconstructions of the central surface in one step (Dahnke et al., 2013). To repair the topological defects, a spherical harmonic method (Yotter et al., 2011a) was used to reparameterize the cortical surface mesh on the basis of an algorithm that reduces area distortions (Yotter et al., 2011c). Then, by quantifying the local fractal dimension, cortical complexity was calculated using the spherical harmonic reconstructions (Yotter et al., 2011b).

Prior to the statistical analyses, the individual cortical thickness and fractal dimension maps were smoothed by using a Gaussian filter with full-width at half-maximum of 15 mm. In addition, vertexwise general linear models were applied to the individual maps, and a multiple regression analysis was performed on the individual cortical thickness and fractal dimension maps. The factor scores were then included as a covariate in the design matrix of SBM analysis. Furthermore, age was included as a nuisance factor in order to correct for the age differences. For the regression analyses, a nonparametric permutation test with 10,000 random permutations was performed. Threshold-Free Cluster Enhancement (TFCE) (Smith and Nichols, 2009) was used to identify the brain regions significantly correlated with each factor score at *p* < 0.05, after correcting for multiple comparisons across space using permutation testing. The anatomical locations of the significant clusters were determined with reference to the Desikan-Killiany atlas (Desikan et al., 2006).

## Results

### Behavioral data

Descriptive statistics for WMS-R subtests were estimated. The item inter-correlation matrix (Table 1) displayed a number of significant correlations and suggested that the WMS-R subtests for patients aged over 75 years may indeed have a structure which could be detected by factor analysis. The number of factors to retain was firstly estimated by the visual inspection of the scree plot (Table 2), which indicates a first break between factors 1 and 2, a second between factors 2 and 3, and a third between 4 and 5. The positions of the breaks suggest three possible solutions: one factor, two factor, and four factor models. We next examined the adequacy of each model by the significance tests obtained from the ML extraction. Chi-square tests were used in order to examine whether each model was sufficient to explain the multivariate relationship of the variables [one factor: $\chi_{\left(54\right)}^{2}$ = 137.80, *p* < 0.0001; two factor: $\chi_{\left(43\right)}^{2}$ = 89.31, *p* < 0.0001; three factor: $\chi_{\left(33\right)}^{2}$ = 56.90, *p* = 0.0060; four factor: $\chi_{\left(24\right)}^{2}$ = 34.65, *p* = 0.074]. On this basis, the four factors appeared to be the most appropriate solution to the model (Table 3). The first factor was defined by visual reproduction I/II and logical memory I/II. We named this factor as “recognition memory.” The second factor was defined by verbal paired associates I/II and visual paired associates I/II. We named this factor as “paired associate memory.” The third factor was defined by visual paired associates I/II, design memory, and digit span. We named this factor as “visual-and-working memory.” The fourth factor was defined by digit span, mental control, and visual span. We named this factor as “attention.”

**Table 1:** Inter-Correlation Matrix for the WMS-R Subtests.

		**Mean ± SD**	**1**	**2**	**3**	**4**	**5**	**6**	**7**	**8**	**9**	**10**	**11**
1	Visual reproduction I	18.6 ± 9.19											
2	Visual reproduction II	5.8 ± 9.11	0.61^*^										
3	Logical memory I	7.9 ± 6.18	0.50^*^	0.54^*^									
4	Logical memory II	2.8 ± 4.72	0.52^*^	0.63^*^	0.78^*^								
5	Verbal paired associates I	7.0 ± 5.66	0.41^*^	0.63^*^	0.64^*^	0.66^*^							
6	Verbal paired associates II	3.0 ± 2.61	0.29	0.59^*^	0.69^*^	0.71^*^	0.87^*^						
7	Visual paired associates I	4.1 ± 4.42	0.38^*^	0.59^*^	0.67^*^	0.69^*^	0.80^*^	0.81^*^					
8	Visual paired associates II	1.8 ± 2.01	0.36^*^	0.65^*^	0.72^*^	0.73^*^	0.74^*^	0.81^*^	0.88^*^				
9	Design memory	4.4 ± 1.85	0.20	0.38^*^	0.37^*^	0.34^*^	0.24	0.28	0.39	0.40^*^			
10	Digit span	10.4 ± 3.35	0.12	0.16	0.16	0.17	0.04	0.06	0.21	0.18	0.12		
11	Mental control	3.1 ± 1.65	0.33^*^	0.13	0.22	0.18	0.21	−0.02	0.17	0.10	−0.10	0.29	
12	Visual span	12.6 ± 3.27	0.42^*^	0.24	0.06	0.20	0.18	0.06	0.25	0.20	0.08	0.37^*^	0.24

*Spearman's ρ-value is shown. The asterisks indicate significant correlations at p < 0.05 (FDR corrected). SD, standard deviation*.

**Table 2:** Total variance explained by different components.

**Component**	**Initial Eigenvalues**			**Extraction sum of squared loading**		
	**Total**	**% of variance**	**Cumulative %**	**Total**	**% of variance**	**Cumulative %**
1	6.02	50.21	50.21	6.02	50.21	50.21
2	1.66	13.83	64.03	1.66	13.83	64.03
3	0.98	8.13	72.16	0.98	8.13	72.16
4	0.91	7.59	79.75	0.91	7.59	79.75
5	0.72	5.98	85.74			
6	0.55	4.61	90.35			
7	0.35	2.91	93.26			
8	0.28	2.34	95.59			
9	0.26	2.13	97.72			
10	0.12	1.03	98.75			
11	0.08	0.71	99.45			
12	0.07	0.54	100.00			

**Table 3:** Four-Factor Promax Solution for WMS-R.

	**Rotated loading**				**Unrotated loading**			
	**RM**	**PM**	**VW**	**AT**	**RM**	**PM**	**VW**	**AT**
Visual reproduction I	**0.76**	0.00	−0.08	0.27	0.53	0.72	−0.23	0.22
Visual reproduction II	**0.84**	0.07	0.08	−0.00	0.62	0.72	−0.15	0.00
Logical memory I	**0.44**	0.25	0.28	−0.04	0.51	0.61	0.08	−0.11
Logical memory II	**0.56**	0.20	0.22	−0.04	0.51	0.61	0.18	−0.13
Verbal paired associates I	0.02	**1.06**	−0.20	0.15	−0.00	1.00	0.00	0.00
Verbal paired associates II	0.08	**0.85**	0.09	−0.22	0.21	0.86	−0.25	−0.23
Visual paired associates I	−0.11	**0.52**	**0.60**	0.17	0.52	0.61	0.41	−0.19
Visual paired associates II	0.19	**0.40**	**0.55**	−0.02	0.45	0.24	−0.09	−0.01
Design memory	0.17	−0.02	**0.44**	−0.07	0.06	0.22	0.38	0.45
Digit span	−0.05	−0.19	**0.47**	**0.36**	0.36	0.49	0.59	0.01
Mental control	0.027	0.07	−0.01	**0.67**	0.18	0.23	0.40	0.44
Visual span	0.12	−0.01	0.08	**0.60**	0.36	0.05	0.07	0.38

*Factor loadings >0.35 were considered significant (shown in **bold**). AT, attention; PM, paired associate memory; RM, recognition memory; SD, standard deviation; VW, visual-and-working memory*.

There were no differences in each factor score between genders (*t*-test, all *p* > 0.25) and no correlations between each factor score and age (Spearman's correlation, all *p* > 0.11), as well as each factor and education (all *p* > 0.09). The total score of the Mini-Mental State Examination (MMSE: 22.8 ± 4.70) positively correlated with the factor scores for recognition memory (ρ = 0.62, *p* < 0.0001), paired associate memory (ρ = 0.42, *p* = 0.0022), and visual-and-working memory (ρ = 0.44, *p* = 0.0013), but not with the factor score for attention (ρ = 0.20, *p* = 0.16). These results reflect the different features of memory-related factors from those of attention factors.

We also examined the difference between factor scores for patients with AD [*n* = 23; age: 82.7 ± 1.05 (75–91); 10 males and 13 females; education (year): 12.0 ± 0.57] and those for patients MCI [*n* = 14; age: 82.9 ± 1.35 (75–92); 5 males and 9 females; education (year): 13.2 ± 0.73]. There were no differences in age (Wilcoxon rank sum test, *p* = 0.96), education (*p* = 0.15), and gender (Chi-square tests: *p* = 0.22). In contrast, there was significant difference in MMSE (*p* < 0.0001). Regarding the factor score, a repeated measures analysis of variance with two factors [disease (AD, MCI) × factor (recognition, paired associate, visual-and-working, attention)] revealed a significant main effect of disease [*F*_(1, 35)_ = 12.72, *p* = 0.0011] with no main effect of factor [*F*_(3, 105)_ = 1.00, *p* = 0.39] nor interaction [*F*_(3, 105)_ = 0.42, *p* = 0.73]. Compared with the patients with AD, the patients with MCI showed significantly higher factor score for paired associate memory [*t*_(35)_ = 3.82, *p* = 0.0005] and visual-and-working memory [paired-*t* test: *t*_(35)_ = 2.79, *p* = 0.0084], and marginally higher factor score for attention [*t*_(35)_ = 2.01, *p* = 0.053]. In contrast, there was no difference in factor score for recognition memory between AD and MCI [*t*_(35)_ = 1.53, *p* = 0.14]. These results suggest the differential features among memory related factors in a memory clinic setting.

### rCBF analysis

rCBF and ρ-values are shown in Table 4. It was found that factor score for paired associate memory positively correlated with rCBF in the left pericallosal and hippocampal regions (Spearman's correlation, FDR corrected *p* < 0.05). The factor score for recognition memory also positively correlated with rCBF in the left pericallosal region (*p* < 0.05). In contrast, the factor scores for visual-and-working memory and for attention showed no significant correlation with rCBF (all, *p* > 0.3).

**Table 4:** Correlation analyses between the factor scores and rCBF.

	**Side**	**Mean ± *SD* (ml/min/100 g)**	**RM**	**PM**	**VW**	**AT**
Callosomarginal	L	37.43 ± 5.89	0.18	0.11	0.25	0.06
	R	37.04 ± 5.77	0.15	0.08	0.21	0.08
Precentral	L	39.31 ± 5.13	0.14	0.11	0.18	0.05
	R	38.72 ± 5.03	0.02	0.02	0.10	0.06
Central	L	38.50 ± 5.65	0.10	0.15	0.10	0.07
	R	37.35 ± 5.25	0.08	0.04	0.08	0.11
Parietal	L	34.00 ± 4.38	0.23	0.25	0.14	−0.02
	R	33.85 ± 4.68	0.20	0.19	0.04	0.13
Angular	L	35.82 ± 4.46	0.08	0.23	0.09	−0.05
	R	36.18 ± 5.13	0.14	0.14	−0.00	0.11
Temporal	L	33.98 ± 4.32	0.09	0.20	0.06	−0.08
	R	33.75 ± 4.24	0.13	0.18	0.01	0.05
Posterior cerebral	L	41.79 ± 5.59	0.06	0.20	0.06	−0.05
	R	41.74 ± 5.22	0.13	0.16	−0.04	0.12
Pericallosal	L	40.21 ± 5.56	0.32^*^	0.37^*^	0.25	0.10
	R	40.32 ± 5.04	0.29	0.33	0.28	0.12
Lenticular nucleus	L	47.90 ± 5.96	−0.10	−0.06	−0.08	−0.01
	R	46.36 ± 6.00	−0.18	−0.08	−0.06	−0.07
Thalamus	L	41.55 ± 5.15	0.15	0.16	0.08	0.07
	R	42.64 ± 5.80	0.18	0.05	0.02	−0.03
Hippocampus	L	31.89 ± 4.37	0.25	0.42^*^	0.22	−0.02
	R	31.16 ± 4.44	0.14	0.35	0.30	−0.06
Cerebellum	L	50.01 ± 9.15	−0.09	0.00	−0.20	−0.00
	R	51.23 ± 8.60	−0.04	0.04	−0.13	0.04

*Spearman's ρ value is shown. The asterisks indicate significant correlations at p < 0.05 (FDR corrected). AT, attention; L, left; PM, paired associate memory; R, right; RM, recognition memory; SD, standard deviation; VW, visual-and-working memory*.

### Cortical thickness analysis

**Figure 1:**
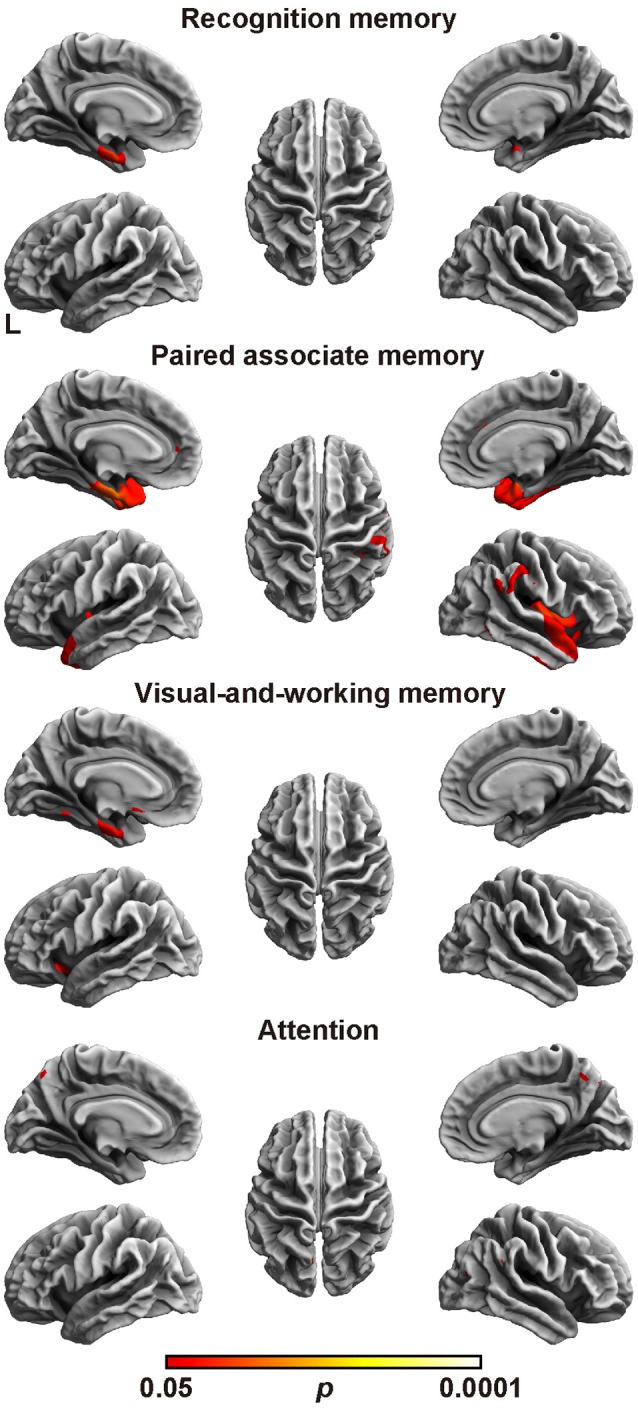
Brain regions with a positive correlation between cortical thickness and factor scores. Significant regions are identified by surface-based morphometry, which were projected onto the left and right lateral surfaces of the standard inflated brain. Medial sections are also shown. The threshold was set at *p* < 0.05 (TFCE-corrected permutation test). See Table 5 for the details of the regions. L, left.

Next, the relation between cortical thickness and each factor score was accessed (Figure 1 and Table 5). The SBM analysis showed that the factor score for paired associate memory most robustly correlated with cortical thickness. This factor score positively correlated with cortical thickness in the limbic system, including the bilateral entorhinal cortex, bilateral insula, and left parahippocampal gyrus. Moreover, it positively correlated with cortical thickness in the widely distributed regions, including the bilateral frontotemporal regions. A less prominent correlation with the cortical thickness in the limbic system was found in the factor scores for recognition memory and visual-and-working memory. In contrast, we found the significant correlation between the factor scores for attention and the cortical thickness in the bilateral precuneus cortex. These results demonstrate the differential neuroimaging features of memory-related factors and attention factor for cortical thickness.

**Table 5:** Cortical thickness correlated with each factor score.

**Brain region**	**Left**				**Right**			
	**RM**	**PM**	**VW**	**AT**	**RM**	**PM**	**VW**	**AT**
Entorhinal cortex	+	+	+			+		
Parahippocampal gyrus	+	+	+					
Temporal pole		+			+	+		
Fusiform gyrus		+	+			+		
Superior temporal gyrus		+				+		
Inferior temporal gyrus		+			+	+		
Superior frontal gyrus						+		
Lateral orbital frontal cortex			+			+		
Medial orbital frontal cortex			+					
Insula		+	+		+	+		
Postcentral gyrus						+		
Supramarginal gyrus						+		
Inferior parietal cortex						+		
Precuneus cortex				+				+

*The threshold was set at p < 0.05 (TFCE-corrected permutation test). AT, attention; PM, paired associate memory; RM, recognition memory; VW, visual-and-working memory*.

### Cortical fractal dimension analysis

**Figure 2:**
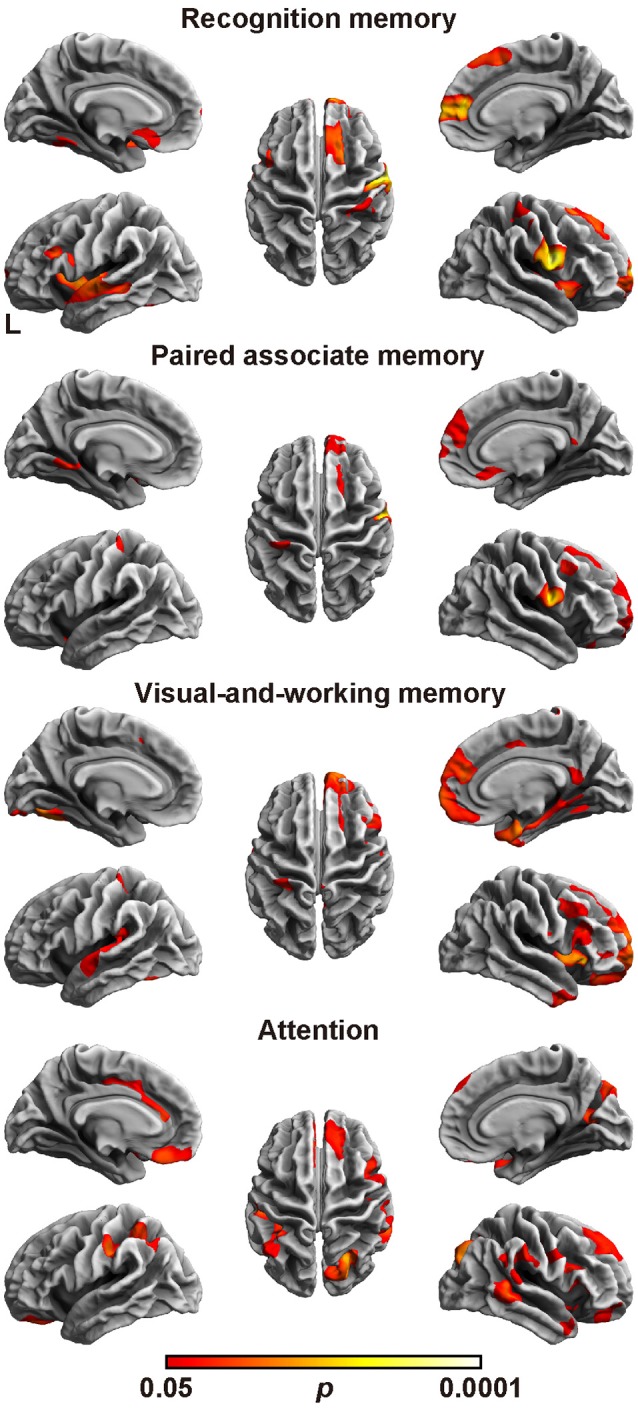
Brain regions with a positive correlation between cortical fractal detention and factor scores. The threshold was set at *p* < 0.05 (TFCE-corrected permutation test). See Table 6 for the details of the regions. L, left.

Finally, we assessed the relation between the cortical fractal dimension and each factor score (Figure 2 and Table 6). The SBM analysis showed that each factor score positively correlated with the fractal dimension in the bilateral frontotemporal regions. Especially, the factor scores for recognition memory most robustly correlated with the fractal dimension in the left frontotemporal regions, whereas the factor scores for visual-and-working memory and attention most prominently correlated with the fractal dimension in the right frontotemporal regions. Interestingly, the factor score for attention selectively correlated with the fractal dimension in the right precuneus cortex, right pericalucarine cortex, and cuneus cortex, which were not correlated with other factor scores. The factor score for visual-and-working memory also showed selective correlations with the fractal dimension of the left lateral occipital cortex. These results demonstrate the distinct neuroimaging features of both attention and visual-and-working memory factors for cortical complexity.

**Table 6:** Cortical fractal dimension correlated with each factor score.

**Brain region**	**Left**				**Right**			
	**RM**	**PM**	**VW**	**AT**	**RM**	**PM**	**VW**	**AT**
Entorhinal cortex							+	
Parahippocampal gyrus		+					+	
Temporal pole							+	+
Fusiform gyrus	+		+				+	
Superior temporal gyrus	+		+		+	+	+	+
Middle temporal gyrus	+						+	+
Inferior temporal gyrus							+	
Transverse temporal cortex	+		+		+			
Banks of the superior temporal sulcus								+
Superior frontal gyrus	+		+	+	+	+	+	+
Rostral middle frontal gyrus	+				+	+	+	+
Caudal middle frontal gyrus						+	+	+
Pars opercularis	+						+	+
Pars triangularis	+						+	+
Pars orbitalis								+
Lateral orbital frontal cortex	+	+		+	+	+	+	+
Medial orbital frontal cortex	+			+		+	+	
Insula	+	+	+		+		+	+
Frontal pole					+	+	+	
Precentral gyrus					+	+	+	+
Paracentral lobule				+				
Postcentral gyrus		+	+	+	+	+	+	+
Supramarginal gyrus			+	+	+	+		+
Superior parietal cortex			+	+	+			+
Inferior parietal cortex				+				+
Precuneus cortex								+
Lingual gyrus		+	+				+	+
Pericalcarine cortex								+
Cuneus cortex								+
Lateral Occipital cortex			+					
Rostral anterior cingulate cortex	+			+	+	+		
Caudal anterior cingulate cortex				+				
Posterior-cingulate cortex							+	
Isthmus-cingulate cortex						+	+	

*The threshold was set at p < 0.05 (TFCE-corrected permutation test). AT, attention; PM, paired associate memory; RM, recognition memory; VW, visual-and-working memory*.

## Discussion

This study demonstrated the four-factor model of the WMS-R in patients aged over 75 years. The model comprised recognition memory, paired associate memory, visual-and-working memory, and attention as factors (Table 3). When compared with the patients with AD, the patients with MCI showed significantly higher factor score for paired associate memory and visual-and-working memory, and marginally higher factor score for attention, whereas there was no difference in factor score for recognition memory between AD and MCI. Regarding the rCBF, the factor score for paired associate memory positively correlated with rCBF in the left pericallosal and hippocampal regions (Table 4). Regarding the cortical thickness (Figure 1 and Table 5), the memory related factor scores correlated with the cortical thickness in the limbic system, in which the factor score for paired associate memory showed most robust correlations, whereas the factor score for attention correlated with the cortical thickness in the bilateral precuneus. Regarding the cortical fractal dimension (Figure 2 and Table 6), each factor score correlated with the bilateral frontotemporal regions, in which the factor score for recognition memory showed most robust correlation with the cortical fractal dimension in the left frontotemporal regions, whereas the factor score for both visual-and-working memory and attention prominently correlated with the cortical fractal dimension in the right frontotemporal regions. Interestingly, the factor scores for the visual-and-working memory and attention selectively correlated with the cortical fractal dimension in the right posterior cingulate cortex and right precuneus cortex, respectively. These findings demonstrate that recognition memory, paired associate memory, visual-and-working memory, and attention can be crucial factors for interpreting the WMS-R results of elderly patients aged over 75 years in a memory clinic setting. Considering these findings, the results of WMS-R in elderly patients aged over 75 years in the memory clinic setting should be cautiously interpreted.

The four-factor model in this study (Table 3) has both a commonality and distinctiveness from the previously proposed models for people aged under 74 years, such as the two-factor model with general memory and attention, the three-factor model with verbal memory, non-verbal memory, and attention, and the three-factor model with attention, immediate memory, and delayed memory (Bornstein and Chelune, 1988; Roid et al., 1988; Burton et al., 1993). The commonality is that there is the attention factor, suggesting that this factor is significant for the WMS-R, regardless of the situation. In contrast, memory-related factors are differently modeled, suggesting that memory-related factors are affected by age. Indeed, previous studies have demonstrated that age affects the performance of the memory-related subtest, which includes visual reproduction, visual paired associates memory, verbal paired associates memory, and logical memory (Cullum et al., 1990; Kawano et al., 2013). The other commonality is the multifaceted nature of subtests. Previous studies have reported the multifaceted nature of some Wechsler subtests such as visual reproduction, visual paired associates, and verbal paired associates (Tulsky and Price, 2003; Duff et al., 2005). In the present study, the visual-paired associates I/II and digit span subtests loaded onto two types of factor, suggesting the multifaceted nature of the WMS-R for elderly patients aged over 75 years in the memory clinic setting. Regarding the clinical practice at a memory clinic setting, previous studies demonstrated that the assessment of a combination of logical memory (Marquis et al., 2002; Rabin et al., 2009) and other cognitive domains, such as visual reproduction, may prove useful for clinical diagnosis in memory clinic setting (Griffith et al., 2006; Tabert et al., 2006; Hori et al., 2013). Although the previous factor analytic studies do not achieve a clear consensus of the factor structure of the WMS-R in clinical populations, it is supposed that among the individual WMS-R subtests, digit span, visual memory, and the logical memory subtests have shown sufficient reliability to be interpreted on their own (Elwood, 1991). These date indicate that logical memory and visual reproduction are distinct factor for patients in the memory clinic setting. The four-factor model in this study also modeled these subtests as one factor. Considering these findings, the effect of each memory-related subtest of WMS-R are varied depending on situation and age, and therefore, the results of WMS-R in elderly patients aged over 75 years in the memory clinic setting should be cautiously interpreted.

The verbal and visual paired associates I/II subtests were modeled as one factor that showed the most prominent correlation with the MMSE score, rCBF, cortical thickness, and cortical fractal dimension in the limbic system and temporal regions (Figures 1, 2 and Tables 3–6). In addition, the patients with MCI showed significantly higher factor score for paired associate memory than patients with AD. These findings suggest that paired associates memory is an important factor for predicting memory problems and cortical abnormalities and for the discrimination between AD and MCI for patients aged over 75 years in a memory clinic setting. Paired associate memory has been repeatedly used to assess declarative memory in previous studies (Kessels et al., 2011; Stollery and Christian, 2015; Papalambros et al., 2017). Declarative memory is mediated by the circuitry involving bidirectional connections between the neocortex, the parahippocampal region, and the hippocampus (Eichenbaum, 2000). Moreover, it is well-documented that declarative memory dysfunction is caused not only in dementia but also in several conditions such as temporal lobe epilepsy (Helmstaedter et al., 1997), schizophrenia (Cirillo and Seidman, 2003), depression (Bremner et al., 2004), and post-traumatic stress disorders (Vermetten et al., 2003), all of which is associated with the dysfunction of hippocampal and temporal regions. These findings suggest that paired associate memory dysfunction can be the most robust indicator of memory problems in elderly patients aged over 75 years.

The visual paired associates I/II, design memory, and digit span were modeled as one factor of visual-and-working memory (Table 3). In addition, the patients with MCI showed significantly higher factor score for this factor than patients with AD, indicating the usefulness in a memory clinic setting for patients over 75 years. Moreover, this factor correlated with the cortical thickness in the limbic system and with the cortical fractal dimension in the right frontotemporal regions (Figure 1 and Table 5). Furthermore, this factor scores selectively correlated with the cortical fractal dimension in the right posterior cingulate cortex (Figure 2 and Table 6). The visual paired associates I/II and design memory is closely linked to the visual memory, whereas the digit span is related to “verbal” working memory (Conway et al., 2005). Therefore, it is difficult to explain this factor by one cognitive aspect. Rather, it should be considered that this factor is associated with the clinical practice for the discrimination between AD and MCI. Visual memory is known to be affected by AD and MCI (Kawas et al., 2003; Barbeau et al., 2004). The working memory is also one aspect of memory affected in the early stages of AD (Belleville et al., 1996; Collette et al., 1999). Regarding neuroimaging data, previous study has suggested that the cortical abnormality in the posterior cingulate cortex is characteristic of AD pathology (Minoshima et al., 1994, 1997; Lehmann et al., 2010). Our results are consistent with these findings and further suggest that the subtests of visual paired associates and figure memory may be useful for the assessment of visual memory for patients aged over 75 years in a memory clinic setting.

The digit span, mental control, and visual span subtests were modeled as one factor of attention (Table 4), which is consistent with previous studies on participants aged under 74 years (Bornstein and Chelune, 1988; Roid et al., 1988; Burton et al., 1993). This indicates that the attention factor is significant in the memory clinic setting, regardless of age. Regarding the neuroimaging findings, the attention factor correlated with the cortical abnormalities in precuneus cortex, which is not correlated with the factor scores for memory-related factors (Figures 1, 2 and Tables 5, 6). Several functional roles for precuneus have been proposed such as attention, visuo-spatial imagery, episodic memory retrieval, self-processing, and consciousness (Cavanna and Trimble, 2006). Especially, a previous positron emission tomography study has demonstrated that a dysfunction in the bilateral precuneus correlated with the severity of autobiographical memory impairment in AD (Eustache et al., 2004). Indeed, the precuneus atrophy is one of diagnostic markers of AD (Karas et al., 2007). Considering these findings, our findings further suggested that the attention factor may be one of the cognitive markers for AD in a memory clinic setting for patients aged over 75 years. Further study is required to confirm the usefulness of this cognitive marker.

The subtests of visual reproduction I/II and logical memory I/II subtests were modeled as a factor of recognition memory (Table 1), which showed less informative neuroimaging features when compared with the other memory-related factors (Figures 1, 2 and Tables 4–6). Recognition memory, a subcategory of declarative memory, is the ability to recognize previously encountered events, objects, or people. It was known that not only visual reproduction memory but also logical memory have been used to assess recognition memory (Perry and Hodges, 2000; Müller et al., 2007; Hori et al., 2013). Thus, this factor mainly reflects recognition memory. Our findings suggest that recognition memory is less informative for the discrimination between AD and MCI for patients aged over 75 years in a memory clinic setting. Regarding the clinical aspect, accumulating evidence from previous studies suggested that a combination of logical memory (Marquis et al., 2002; Rabin et al., 2009) and other cognitive domains, such as visual reproduction, may prove useful for detecting MCI (Griffith et al., 2006; Tabert et al., 2006; Hori et al., 2013). The present findings may reflect that recognition memory is sensitive to MCI but not to the difference between MCI and AD. Regarding the neuroimaging data, a previous study demonstrated that the correlation between these subtests and the hippocampal volume on MRI was relatively weak for participants comprising AD patients and control subjects (Petersen et al., 2000), which is consistent with the present findings. It is also known that these subtests negatively correlated with age (Haaland et al., 2003). Although the effect of age was factored out in the neuroimaging analysis in our study (which may affect the results of our study), the findings support the conclusion that the results of the subtests related to recognition memory should be cautiously interpreted in the memory clinic setting for patients aged over 75 years.

This study has some limitations. First, patients with heterogeneous profiles were allowed to participate in the present study. In other words, as it aimed to assess the usefulness of the WMS-R in a memory clinic setting, all the participants who met the inclusion criteria (see section Materials and Methods) were included, regardless of their profiles. Certainly, disease pattern was consistent with that of a previous study in a memory clinic setting (Wada-Isoe et al., 2009). Second, as this study utilized a relatively small sample size, the significance of recognition memory remains unclear. In the previous studies, the same subtests regarding visual reproduction and logical memory were used to assess patients with different profiles, such as those with AD, Huntington disease, or temporal lobe epilepsy (Troster et al., 1993; Lacritz et al., 2004). Therefore, future studies should clarify the factor structure of the WMS-R and its neuroimaging features for specific neurological profiles.

In conclusion, recognition memory, paired associate memory, visual-and-working memory, and attention can be crucial factors for interpreting the WMS-R results of elderly patients aged over 75 years in a memory clinic setting. The WMS-R requires more time to complete, which may be difficult for elderly patients. Nevertheless, administering the entire WMS-R, including paired associate memory, can be useful for assessing elderly patients with memory problems.

## Author contributions

RK and AS: contributed to the conception and design of this research; RK: in particular, contributed to the analysis and interpretation of the data as well as the initial drafting of the work. All authors contributed to the acquisition of the data, critically revised it for important intellectual content, and approved its final version. All authors are accountable for the contents of this research.

### Conflict of interest statement

The authors declare that the research was conducted in the absence of any commercial or financial relationships that could be construed as a potential conflict of interest.
